# Mutational landscape and risk estimates of DDR genes in Chinese ovarian cancer patients

**DOI:** 10.1186/s13048-025-01925-7

**Published:** 2025-12-10

**Authors:** Cuiyun Zhang, Bing Wei, Xia Xue, Qingxin Xia, Yi Wang, Lanwei Guo, Tingjie Wang, Li Wang, Junli Deng, Yuping Guan, Xiaoyan Wang, Lu Feng, Rui Wu, Ziqing Hu, Klaas Kok, Anke van den Berg, Yongjun Guo, Jun Li

**Affiliations:** 1 Department of Molecular Pathology, The Affiliated Cancer Hospital of Zhengzhou University and Henan Cancer Hospital, No.127 Dongming Road, Zhengzhou, 450008 China; 2 Henan Key Laboratory of Molecular Pathology, Zhengzhou, China; 3 Henan International Joint Laboratory of Cancer Genetics, Zhengzhou, China; 4 Henan Key Laboratory for Helicobacter pylori and Digestive Tract Microecology, The Fifth Affiliated Hospital of Zhengzhou University, Zhengzhou, China; 5 Institute of Rehabilitation Medicine, Henan Academy of Innovations in Medical Science, Zhengzhou, China; 6 Department of Pathology, The Affiliated Cancer Hospital of Zhengzhou University and Henan Cancer Hospital, Zhengzhou, China; 7 Department of Clinical Research Management, The Affiliated Cancer Hospital of Zhengzhou University and Henan Cancer Hospital, Zhengzhou, China; 8 Department of Gynecologic Oncology, The Affiliated Cancer Hospital of Zhengzhou University and Henan Cancer Hospital, Zhengzhou, China; 9 Department of Head and Neck Surgery, The Affiliated Cancer Hospital of Zhengzhou University and Henan Cancer Hospital, Zhengzhou, China; 10 Department of Pathology and Laboratory Medicine, Memorial Sloan Kettering Cancer Center, New York, USA; 11 Department of Applied and Computational Mathematics and Statistics, University of Notre Dame, South Bend, IN USA; 12 Department of Genetics, University of Groningen, University Medical Center Groningen (UMCG), Groningen, the Netherlands; 13 Department of Pathology and Medical Biology, University of Groningen, University Medical Center Groningen (UMCG), Groningen, the Netherlands

**Keywords:** Ovarian cancer, Genetic predispositions, Han Chinese, Risk estimate, DNA damage response genes

## Abstract

**Background:**

Pathogenic/likely pathogenic variants (P/LPVs) in DNA damage response (DDR) genes are known ovarian cancer (OC) risk factors, but gene-specific risk estimates in Han Chinese remain unclear.

**Objective:**

To accurately assess the risk associated with DDR genes in the Han Chinese population to facilitate personalized risk management and enhance clinical decision-making.

**Methods:**

We performed next-generation sequencing of 45 DDR genes in 666 OC patients from Henan, China. Associations between P/LPVs and clinical features were assessed using chi-squared tests. Variant frequencies were compared with population controls (gnomAD and ChinaMAP databases) to estimate gene-specific odds ratios (ORs) using Fisher’s test.

**Results:**

In Henan Ovarian Cancer patients, the median disease onset age was 53 years (range: 24–81), with 7.7% diagnosed before 40. Most patients had advanced disease (56.5% Stage III, 18.9% Stage IV), and 75.8% had high-grade serous carcinoma (HGSC). P/LPVs in *BRCA1/2* were significantly associated with the HGSC subtype and a positive family history (*p* < 0.001 for both). Beyond *BRCA1* (OR = 125.5/146.1) and *BRCA2* (OR = 17.9/20.2), significantly elevated ovarian cancer risks were observed for *RAD51D*, *RAD51C*, and *MSH2* (OR: 10.1–35.4, all *p* < 0.05).

**Conclusions:**

This study provides the first gene-specific ovarian cancer risk estimates for DDR genes in Han Chinese, expanding the high-risk gene spectrum. By defining population-specific risk magnitudes, our findings provide a framework for clinical risk stratification, and underscore the need to tailor screening and prevention strategies to China’s distinct genetic landscape.

**Supplementary Information:**

The online version contains supplementary material available at 10.1186/s13048-025-01925-7.

## Background

Ovarian cancer (OC) is the third most common gynecological malignancy characterized by a high mortality rate. The five-year survival rate for OC is highly stage-dependent, ranging from 15% in stage IV disease to nearly 95% in stage I. Unfortunately, over 70% of patients are diagnosed at advanced stages [1, 2]. In 2022, the estimated global incidence of OC was 324,398 cases, with 61,100 cases reported in China [3, 4]. Standard screening methods, including transvaginal ultrasound and CA-125 blood tests, have been shown to be ineffective in reducing the incidence of OC or improving survival outcomes [5], therefore, targeted management of high-risk individuals may provide a more efficient strategy to reduce both the incidence and mortality of OC.

Carriers of pathogenic/likely pathogenic variants (P/LPVs) in *BRCA1* have a 20–30 times higher lifetime OC risk compared to the general population, while those with *BRCA2* P/LPVs increased approximately 10–20 times [6, 7]. Other genes implicated with elevated OC risk include *BRIP1*, *PALB2*, *RAD51C*, *RAD51D*, and *STK11*, most of which are involved in DNA damage response (DDR) [8, 9]. Accurate risk assessment is crucial for managing the carriers of OC predisposition genes, particularly in facilitating informed risk-reducing bilateral salpingo-oophorectomy (RRSO) decisions that should balance between benefits and risks [10]. However, precise risk estimates of these genes to OC have not yet been determined in the Chinese population. This study aims to determine the precise risk estimates of DDR genes in Han Chinese to support tailored risk management strategies and improved clinical decision-making.

## Methods

### Study cohort

The patients enrolled in this study were admitted to Henan Cancer Hospital between April 2018 and December 2021 with a confirmed diagnosis of epithelial ovarian cancer by two independent pathologists. Those without testing for germline variants were excluded from this analysis. To minimize potential confounding from population stratification, we restricted our analysis to a highly homogeneous patient population, of whom 98.8% (658/666) self-identified as Han Chinese. For comparison, the China Metabolic Analytics Project (ChinaMAP) reference database, which includes approximately 85.4% (9,043/10,588) Han Chinese individuals, was used alongside the East Asian subset of the Genome Aggregation Database (gnomAD) to ensure close ancestral matching with our predominantly Han Chinese cohort. We did not perform principal component analysis (PCA) or adjust for genetic ancestry because individual-level genotypic and phenotypic data were not available for the gnomAD and ChinaMAP reference populations. This study was approved by the Ethics Committee of Henan Cancer Hospital (approval number: 2021-KY-0091-002), and written informed consent was obtained from all participants in accordance with the Declaration of Helsinki.

### DNA extraction, targeted sequencing, variant calling, and classification

Genomic DNA was extracted from the leukocyte fraction of 500µL peripheral blood using the QIAamp Blood DNA isolation kit (Qiagen, Germany) to minimize circulating tumor DNA (ctDNA) contamination, then quantified by Qubit dsDNA HS assay (Life Technologies, USA). A total of 200ng DNA was subjected to next-generation sequencing library construction following optimized protocols as previously described [11]. Coding exons and flanking intronic regions (± 20 bp) of target genes were polymerase chain reaction (PCR)-amplified using the DDR 45-gene kit (Novogene Biotech, China). Investigated genes are listed in Table S1. Indexed samples were sequenced with 121 bp paired-end reads on a NextSeq 550 platform (Illumina, USA). Reads were aligned to the hg19 reference genome by Burrows-Wheeler Aligner (bwa-mem v0.7.17) [12]. BAM files were sorted, and duplicated reads were marked and removed using SAMtools (v1.9.0) [13], sambamba (v0.7.1) [14]. Germline variants were called by three independent algorithms: Genome Analysis Toolkit Haplotype joint caller (GATK-haplotype v4.1.7.0) [15], FreeBayes (v1.1.0.46) [16], and SAMtools (v1.9.0) [13]. Called variants were filtered using strict thresholds (minimum depth 100× and variant allele frequency ≥ 30%, variant quality ≥ 30), followed by annotation with snpEff software (v4.3.1t) [17]. Randomly selected P/LPVs were Sanger-validated using leukocyte-derived DNA. Variants were described according to the Human Genome Variation Society (HGVS) nomenclature and annotated using public databases, including ClinVar, gnomAD, dbSNP, and COSMIC, to help identify known germline variants and exclude likely somatic mutation hotspots. Subsequently, variants were classified by two geneticists independently using the five-tiered system proposed by the American College of Medical Genetics and Genomics (ACMG) and the Evidence-based Network for the Interpretation of Germline Mutant Alleles (ENIGMA).

### Statistical analysis and risk estimates

Categorical variables, including disease onset age, disease stage, histological subtypes, and family history, were compared across *BRCA1/2*^+^ (with P/LPVs in *BRCA1* or *BRCA2* genes), *DDR*^+^ (with P/LPVs in non-*BRCA1*/*2* DDR genes), and *DDR*^-^ (negative for any DDR P/LPVs) patients using the chi-squared test. Odds ratios (ORs) and 95% confidence intervals (CIs) for each gene were calculated by Fisher’s exact test. Risk estimates for each gene were determined by comparison with their prevalence in the East Asian population of gnomAD (v4.1.0, https://gnomad.broadinstitute.org) and ChinaMAP (v2020-03.beta, www.mbiobank.com) [18, 19]. To correct for multiple hypothesis testing while preserving statistical interpretability, Benjamini-Hochberg false discovery rate (FDR) correction was applied only to genes observed in ≥ 2 Henan ovarian cancer (HOC) patients with a nominal *p*-value < 0.1 in either comparison. All statistical analyses were conducted using R software (v4.3.1), and two-sided *p*-values < 0.05 were considered statistically significant.

## Results

### Clinical characteristics of the patients

The median age at disease onset among HOC patients was 53 years (range: 24–81). No significant differences in age at disease onset were observed across subgroups: *BRCA1/2*^+^ patients had a median onset age of 52 years (range: 33–80), *DDR*^+^ patients had a median age of 53 years (range: 33–75), and *DDR*^−^ patients had a median age of 54 years (range: 24–81). Early-onset disease (< 40 years) was observed in 7.7% (51/666) of patients, with no significant difference among the *BRCA1/2*^*+*^, *DDR*^*+*^, and *DDR*^*−*^ patients (Table 1). In terms of disease stages, 10.2% (68/666) of patients were diagnosed at stage I, 6.9% (46/666) at stage II, 56.5% (376/666) at stage III, 18.9% (126/666) at stage IV, and 7.5% (50/666) with unknown stage status. Chi-squared analysis revealed a significant difference in stage distribution between *BRCA1/2*^*+*^ and *DDR*^*−*^ patients (*p* = 0.0084), notably with stage I disease being less frequent in *BRCA1/2*^*+*^ patients, suggesting a potential gap in the implementation of targeted screening and risk management for *BRCA1/2*^*+*^ carriers in this cohort.

**Table 1:** Clinical characteristics of Henan Ovarian Cancer (HOC) patients included in this study

Characteristics	Total (*n* = 666)		*BRCA1/2*^*+*^ (*n* = 181)		*DDR*^*+*^ (*n* = 50)		*DDR*^*−*^ (*n* = 444)		*p*-value		
	cases	%	cases	%	cases	%	cases	%	BRCA1/2^+^ vs. DDR^+^	BRCA1/2^+^ vs. DDR^−^	DDR^+^ vs. DDR^−^
<40 (years old)	51	7.66%	9	4.97%	3	6.00%	39	8.78%	1.0	0.14	0.69
≥40 (years old)	615	92.34%	172	95.03%	47	94.00%	405	91.22%			
Stage											
I	68	10.21%	8	4.42%	6	12.00%	54	12.16%	0.098	0.0084	0.22
II	46	6.91%	19	10.50%	2	4.00%	26	5.86%			
III	376	56.46%	100	55.25%	23	46.00%	258	58.11%			
IV	126	18.92%	37	20.44%	15	30.00%	75	16.89%			
Unknown	50	7.50%	17	9.39%	4	8.00%	31	6.98%			
Histological Subtypes											
High Grade Serous	505	75.83%	156	86.19%	36	72.00%	321	72.30%	3.26e-07	1.04e-05	0.38
Low Grade Serous	20	3.00%	1	0.55%	0	0.00%	19	4.28%			
Endometrioid	23	3.45%	0	0.00%	3	6.00%	20	4.50%			
Clear cell	22	3.30%	0	0.00%	4	8.00%	18	4.05%			
Mucinous	18	2.70%	0	0.00%	3	6.00%	15	3.38%			
Unspecified	78	11.72%	24	13.26%	4	8.00%	51	11.49%			
Family history											
Yes	49	7.36%	30	16.57%	4	8.00%	16	3.60%	0.20	4.66e-08	0.26
No	617	92.64%	151	83.43%	46	92.00%	428	96.40%			

Chi-squared test was used to compare clinical characteristics between groups. A *p*-value of < 0.05 was considered as statistically significant. *BRCA1/2*^*+*^ patients are the ones with *BRCA1* or *BRCA2* P/LPVs; *DDR*^*+*^ patients are the ones with germline P/LPVs in non-*BRCA1/2* DDR genes; *DDR*^*−*^ patients are the ones without any P/LPVs in DDR genes

Regarding histological subtypes, high-grade serous carcinoma (HGSC) was the predominant subtype, accounting for 75.8% (505/666) of the cases. Other subtypes included endometrioid (3.5%, 23/666), clear cell (3.3%, 22/666), low-grade serous ovarian cancer (3.0%, 20/666), and mucinous carcinoma (2.7%, 18/666), with 11.7% (78/666) cases unspecified. *BRCA1/2*^*+*^ patients showed a significant enrichment of HGSC compared to the *DDR*^*+*^ (*p* = 3.26e-07) and *DDR*^*−*^ (*p* = 1.04e-05) groups, except for one *BRCA2*^*+*^ patient presenting with low-grade serous ovarian cancer. Additionally, a family history of cancer was reported in 49 of the 666 patients (7.4%), with *BRCA1/2*^*+*^ patients showing a significantly higher likelihood compared to *DDR*^*-*^ patients (*p* = 4.7e-08).

### Genetic predispositions in DDR genes

Each sample generated approximately 3 Mb of high-quality reads, with an average coverage exceeding 500×. Over 99% of the targeted regions achieved a coverage depth greater than 100×. A total of 232 P/LPVs spanning 21 genes were identified in 222 of the 666 patients. Notably, 12.9% (30/232) of these variants were previously unreported in ClinVar or other public databases (Table S2). The identified P/LPVs consisted of 142 frameshift, 57 stop-gain, 12 missense, 19 splice-site, and 2 start-codon variants.

Among the 19 splice-site variants, 14 were located at canonical acceptor/donor sites, 3 at intronic boundaries, and 2 at exon ends. One notable variant, *BRCA1*:c.132 C > T (p.Cys44=), a synonymous variant at the exon3 boundary, was detected in two unrelated individuals. Functional analyses revealed that this synonymous change disrupts protein function by inducing aberrant splicing [20].

**Fig. 1:**
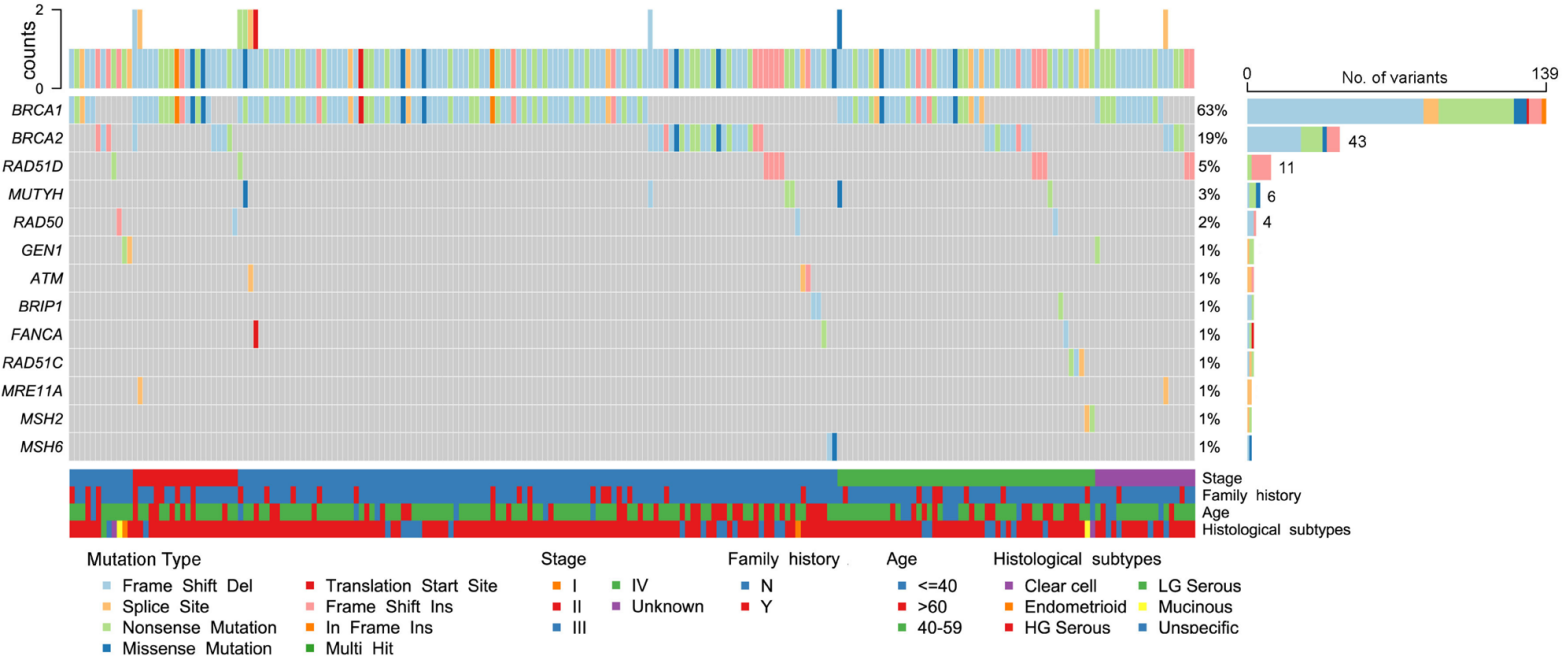
Genetic predispositions of pathogenic or likely pathogenic (P/LPVs) in Henan ovarian cancer (HOC) patients. Oncoplot presents the distribution of germline P/LPVs detected in ≥ 2 independent individuals of this study. The upper panel shows the number and subtype of PVs/LPVs detected in each patient. The middle panel displays the number of P/LPVs detected in each gene and its proportion among the total variants (*n* = 232). Clinical characteristics of the patients are indicated by different colors and clustered according to disease stages

The top five mutants identified in HOC patients were *BRCA1* (*n* = 139), *BRCA2* (*n* = 43), *RAD51D* (*n* = 11), *MUTYH* (*n* = 6), and *RAD50* (*n* = 4), accounting for 59.9%, 18.5%, 4.7%, 2.5%, and 1.7% of all detected variants, respectively. Additional mutations were identified in *GEN1* (*n* = 3), *ATM* (*n* = 3), *BRIP1* (*n* = 3), *FANCA* (*n* = 3), *RAD51C* (*n* = 3), *MRE11A* (*n* = 2), *MSH2* (*n* = 2) and *MSH6* (*n* = 2) (Fig. 1). P/LPVs in *FANCL*, *FANCM*, *PALB2*, *TP53*, *ATR*, *CHEK1*, *ERCC3*, and *RAD54L* were each detected in only one patient. In general, most P/LPVs were mutually exclusive; however, ten patients harbored dual variants, consistently involving at least one in *BRCA1* or *BRCA2* (Table S3). This underscores the critical role of *BRCA1/2* as key drivers in ovarian cancer pathogenesis.

## Risk estimates of DDR genes in the HOC cohort

As expected, *BRCA1* and *BRCA2* demonstrated the strongest associations with ovarian cancer risk in our cohort. When compared with gnomAD, *BRCA1* P/LPVs showed a significantly increased OR of 125.5 (95%CI: 88.7–180.1, *p*-adj = 2.0e-176), while *BRCA2* P/LPVs had an OR of 17.9 (95%CI: 12.0–26.4, *p*-adj = 2.3e-33). A similar trend was also observed when compared with ChinaMAP database, where *BRCA1* and *BRCA2* P/LPVs showed ORs of 146.1 (95% CI: 89.4–253.1, *p*-adj = 7.6e-153) and 20.2 (95%CI: 12.5–32.6, *p*-adj = 1.2e-31), respectively.

Although less frequent, P/LPVs in *RAD51D*, *RAD51C*, and *MSH2* also showed significantly increased ORs (OR > 10 and *p* < 0.05 for all) when compared with both the gnomAD and ChinaMAP databases. *MUTYH*, however, only exhibited significantly increased ORs when compared with the gnomAD database, as detailed in Table 2. For other DDR genes—including *BRIP1*, *ERCC3*, *RAD54L*, *FANCL*, *TP53*, *MRE11A*, *FANCM*, *MSH6*, *RAD50*, *ATM*, and *FANCA*—the ORs exceeded 2, but their associations with OC risk did not reach statistical significance (Table 2 and Table S4). This is likely due to their low variant prevalence and the limited sample size, which precluded precise risk estimation for these rare mutations in this study.

**Table 2:** Genes associated with increased ovarian cancer risk identified in Henan Ovarian Cancer (HOC) cohort

Gene list	HOC (*n* = 666)		gnomAD v4.1.0 East Asian(*n* = 22,448)						ChinaMAP v2020-03.beta (*n* = 10,588)					
	cases	%	cases	%	OR	95% CI	*p*-value	*p*-adj	cases	%	OR	95% CI	*p*-value	*p*-adj
*BRCA1*	139	20.87%	47	0.21%	125.5	88.7–180.1	2.2e-177	2.0e-176	19	0.18%	146.1	89.4–253.1	8.4e-154	7.6e-153
*BRCA2*	43	6.46%	86	0.38%	17.9	12.0–26.4	5.1e-34	2.3e-33	36	0.34%	20.2	12.5–32.6	2.6e-32	1.2e-31
*RAD51D*	11	1.65%	33	0.15%	11.4	5.2–23.3	3.4e-08	1.0e-07	5	0.05%	35.4	11.3–130.2	9.9e-11	3.0e-10
*RAD51C*	3	0.45%	10	0.04%	10.1	1.8–39.6	5.5e-03	0.01	3	0.03%	15.9	2.1–119.4	3.6e-03	8.2e-03
*MSH2*	2	0.30%	6	0.03%	11.3	1.1–63.1	0.02	0.03	2	0.02%	15.9	1.2–219.1	0.02	0.04
*MUTYH*	6	0.90%	62	0.28%	3.3	1.2–7.6	0.01	0.02	44	0.42%	2.2	0.8–5.1	0.1	0.1
*BRIP1*	3	0.45%	27	0.12%	3.8	0.7–12.3	0.05	0.1	16	0.15%	3.0	0.6–10.5	0.1	0.2
*RAD50*	4	0.60%	48	0.21%	2.8	0.7–7.7	0.06	0.07	32	0.30%	2.0	0.5–5.6	0.2	0.2
*MRE11A*	2	0.30%	17	0.08%	4.0	0.4–16.8	0.1	0.1	5	0.05%	6.4	0.6–38.9	0.06	0.09

Fisher’s exact test was applied to determine relative risk for each gene by comparing to its prevalence in the HOC cohort to that in gnomAD (East Asian, v4.1.0) and ChinaMAP databases (v2020-03.beta). False discovery rate (FDR)-adjusted p-values were calculated using the Benjamini-Hochberg procedure. A *p*-value of < 0.05 was considered as statistically significant. OR: odds ratio; 95% CI: 95% confidence interval; *p*-adj: adjusted *p*-value

## Discussion

In this study, we analyzed germline variants in 45 DDR genes among 666 Han Chinese OC patients from Henan, China, identifying P/LPVs in 33.3% (222/666) of the cases. *BRCA1*, *BRCA2*, *RAD51D*, *RAD51C* and *MSH2* were confirmed as high-risk OC predisposition genes in Han Chinese (OR > 10, *p* < 0.05 for all). The prevalence of *DDR* gene P/LPVs in our cohort is higher than that reported in North American and European patients (20.5%–27.5%, Table S5) [21–23]. While the overall distribution of P/LPVs identified in this study largely mirrors patterns observed in other populations, there are some notable differences. For instance, mismatch repair gene variants were limited to *MSH2* and *MSH6* (0.6% vs. 1–2% in other studies) [21–26], likely due to the small number of patients with endometrioid carcinoma (*n* = 23).

Notably, *BRCA1* contributed predominantly to the elevated prevalence of DDR gene predispositions observed in our HOC cohort. While established factors such as ethnicity, histologic subtype, and family history are known to influence *BRCA1* prevalence, they do not fully account for the higher rate observed in our HGSC patients (31%, 156/505), which exceeds previous reports of up to 25% [22, 27, 28]. A review of domestic studies revealed substantial heterogeneity in *BRCA1* prevalence, even among studies conducted by the same research group (Table S6) [11, 29–32], suggesting the presence of population-specific influences. Further analysis revealed a higher frequency of recurrent variants (RVs)—defined as the same P/LPV identified in three or more patients—indicating a potential founder effect (Table S7). This was supported by short tandem repeat (STR) analysis [11, 33], which showed that patients harboring RVs frequently shared identical STR haplotypes flanking the *BRCA1* locus (Fig. S1). For instance, individuals with *BRCA1*:c.5470_5477del shared haplotypes D17S1320 (172) and D17S1327 (129), while those with *BRCA1*:c.5521delA shared D17S846 (237) and D17S1789 (193).

Although no significant difference in disease onset age was observed among *BRCA1/2*^+^, *DDR*^+^, and *DDR*^−^ patients (Table 1), cumulative incidence curves revealed that *BRCA1*^+^ patients experienced significantly earlier disease onset than the others (*p* < 0.01 for all; Fig. S2A), consistent with previous reports [34, 35]. Interestingly, *DDR*^−^ patients exhibited a higher incidence rate than *BRCA1/2*^+^ and *DDR*^+^ patients before the age of 40, suggesting that additional factors may contribute to early-onset disease in this subgroup.

The *Androgen Receptor* (*AR*) gene, expressed in all OC subtypes, harbors a CAG-repeat in exon1 that has been associated with disease onset age in breast and ovarian cancer [36, 37]. In HOC cohort, we identified 28 distinct *AR*-CAG alleles varying in size between 15 and 29 repeats, with 22-repeat being dominant that 88% (585/666) were *AR*-CAG^22/22^ homozygotes (Fig. S2B). Disease incidence curves showed that non-CAG^22/22^ patients were more likely to develop OC before age 40 (*p* = 0.0129, Fig. S2C). This trend was also observed in *BRCA1/2*^−^ patients (Fig. S2D and E), suggesting a *BRCA1/2-*independent effect. Prior studies on CAG-repeat length yielded inconsistent results [37–39]; it remains to be clarified whether disease risk is linked to repeat length or genotype. Of note, no similar trend was observed for the GCG-repeat at the same *AR* exon (Fig. S2F and G).

Our findings on high-risk OC genes (*BRCA1/2*, *RAD51C/D*, and *MSH2;* OR > 10 and *p* < 0.05) generally align with Western studies but differ for moderate- to low-risk genes. For instance, although *MUTYH* showed significance in comparison with gnomAD, its clinical relevance remains uncertain, as half of the six carriers also harboured *BRCA1/2* PVs. Similarly, although *BRIP1* showed borderline significance when compared with the gnomAD database, the association was no longer statistically significant after Benjamini–Hochberg correction for multiple testing. The lack of statistical significance for other moderate- to low-risk genes is likely attributable to the limited sample size of our HOC cohort, which may have reduced the power to detect modest effect sizes. Larger studies or pooled analyses will be necessary to clarify the contributions of these genes to ovarian cancer risk in our population. These results underscore the need for caution when interpreting moderate- to low-risk genes, as genetic susceptibility may be highly context-dependent [21, 22, 40–43].

The NCCN v2.2025 guideline list ten OC risk genes, including *BRCA1/2*, *RAD51C/D*, *BRIP1*, *PALB2*, *MLH1*, *MSH2*, *EPCAM*, and *MSH6*. However, UK experts recently noted that OC risk associated with *RAD51C/D*, *BRIP1*, and *PALB2* strongly depends on family history, highlighting the need for personalized genetic counseling [44, 45]. In contrast, current Chinese guidelines still lack population-based risk evidence, which limits the accuracy and applicability of genetic risk assessment [46, 47]. There is an urgent need for Chinese-specific frameworks to support evidence-based counseling and preventive strategies.

This is the first study providing precise risk estimates for DDR genes in a relatively homogeneous Han Chinese cohort from central China, reducing geographic and ethnic confounding. This homogeneity enabled the identification of a strong *BRCA1* founder effect, which partially explains the high *BRCA1* prevalence in our cohort. However, limitations of our study include single-center design and small sample size, which restrict conclusions for moderate- to low-risk genes, e.g., *MUTYH* and *BRIP1.* Also, our panel (45 genes) did not detect large genomic rearrangements, possibly underestimating variant prevalence [48, 49]. Although formal gene burden testing was not performed, we examined genotype–phenotype associations within the HOC cohort by comparing clinical features across *BRCA1/2*^*+*^, *DDR*^+^, and *DDR*^−^ groups. This group-based comparison—conducted using chi-squared tests as described in the Methods section—provided a preliminary overview of potential genotype-related clinical differences. However, individual gene-level burden testing was not feasible due to the low number of carriers in most non-*BRCA* genes. The analysis requires larger cohorts to achieve adequate statistical power. Future studies with expanded patient numbers or collaborative meta-analyses will be essential to validate gene–phenotype associations more comprehensively.

Recognition of the *BRCA1* founder effect in the Han Chinese population provides an opportunity to streamline genetic screening through targeted testing of recurrent variants. This approach can significantly reduce costs and improve feasibility for large-scale screening programs, particularly in resource-limited regions. In this context, our identification of recurrent variants such as *BRCA1*:c.5470_5477del supports the design of population-specific, first-tier panels tailored to Han Chinese patients. Moreover, our gene-specific risk estimates reveal striking ancestry-related differences. For example, *RAD51D* showed an OR of 35.4 in the Han Chinese cohort (vs. 6–12 reported in Western populations [44]), highlighting the importance of ethnicity-specific risk stratification. These findings argue against the direct application of Western-derived risk estimates to Chinese populations and underscore the need to develop China-specific clinical guidelines for genetic counseling and risk-reducing interventions. However, we also caution that our findings may not generalize to non-Han ethnic groups within China (e.g., Tibetan, Uyghur, Mongolian), for whom genomic data remain scarce. Without ancestry-matched reference data and founder variant characterization in these populations, extrapolation from Han Chinese data is inappropriate. We strongly advocate for inclusive, multi-ethnic research efforts to fill these knowledge gaps and support equitable clinical decision-making across China’s diverse population.

## Conclusion

*BRCA1/2*, *RAD51C/D*, and *MSH2* are high-risk OC predisposition genes identified in Han Chinese.

## Supplementary Information

Supplementary Material 1.

Supplementary Material 2.

## Data Availability

Due to China’s policies on the management of human genetic resources, the datasets generated and analyzed during the current study are not publicly available, but are available from the corresponding author on reasonable request.

## Abbreviations

ACMG: American College of Medical Genetics
ChinaMAP: the China Metabolic Analytics Project
CI: Confidence interval
DDR: DNA damage response
ENIGMA: Evidence-based Network for the Interpretation of Germline Mutant Alleles
gnomAD: the Genome Aggregation Database
HGSC: High-grade serous carcinoma
HGV: Human genome variation society
MMR: Mismatch repair
NGS: Next-generation sequencing
OC: Ovarian cancer
OR: Odds ratio
P/LPVs: Pathogenic or likely pathogenic variants
RRSO: Risk-reducing bilateral salpingo-oophorectomy
RV: Recurrent variants
STR: Short tandem repeat
