# Supplementary material for: Mutational landscape and risk estimates of DDR genes in Chinese ovarian cancer patients
Source: J Ovarian Res. 2025 Dec 10;19:49. doi: 10.1186/s13048-025-01925-7 (PMC12879432; doi:10.1186/s13048-025-01925-7)
Supplement: Supplementary file 1 — Supplementary Material 1. [file 13048_2025_1925_MOESM1_ESM.docx]

**Supplementary Information for *Mutational landscape and risk estimates of DDR genes in Chinese ovarian cancer patients***

**Figure S1.** Strong founder effect in Henan ovarian cancer (HOC) patients with recurrent pathogenic or likely pathogenic variants (P/LPVs).

**Figure S2.** Disease onset age and cumulative analysis incidence by *AR*-CAG and *AR*-GCG polymorphisms in Henan ovarian cancer (HOC) patients.

**Table S1.** Genes included in the DNA damage response (DDR)-targeted panel of this study.

**Table S2.** Total of 232 pathogenic or likely pathogenic variants (P/LPVs) in DNA damage response (DDR) genes.

**Table S3.** Clinical characteristics of Henan ovarian cancer (HOC) patients with double pathogenic or likely pathogenic variants (P/LPVs).

**Table S4.** Ovarian cancer risk estimates for other DNA damage response genes in the Henan ovarian cancer (HOC) cohort.

**Table S5.** Prevalence of ovarian cancer predisposition genes across different populations.

**Table S6.** Clinical characteristics of patients from previous Chinese studies on genetic predisposition to ovarian cancer.

**Table S7.** Recurrent *BRCA1* pathogenic or likely pathogenic variants (P/LPVs) identified in studies of Chinese ovarian cancer patients.


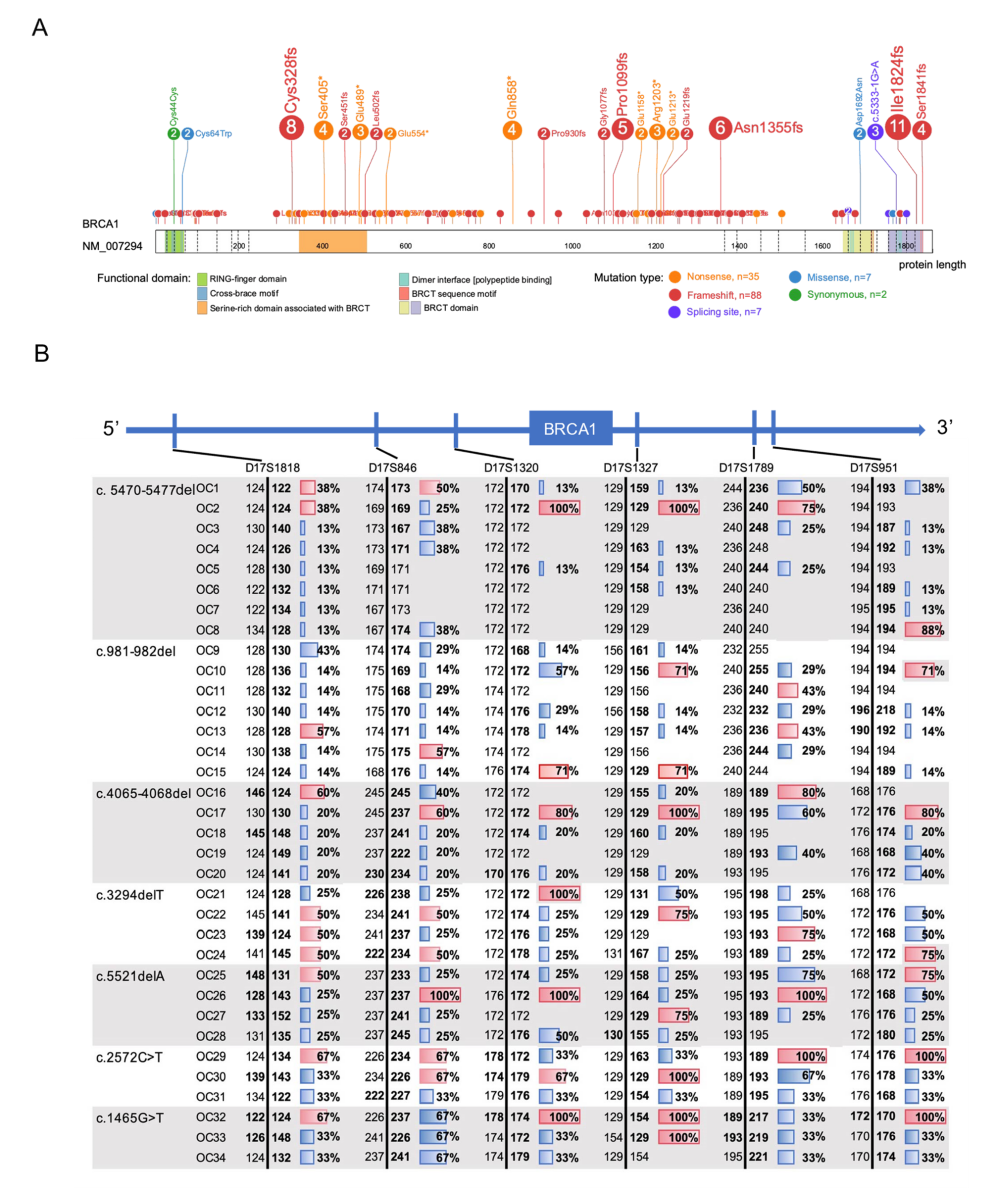


**Figure S1.** Strong founder effect in Henan ovarian cancer (HOC) patients with recurrent pathogenic or likely pathogenic variants (P/LPVs).

**(A)** *BRCA1* P/LPVs detected in HOC cohort. Lollipop chart showed the distribution of all 139 P/LPVs in *BRCA1* detected in HOC patients. Function domains of *BRCA1* and mutation types were indicated in different colors. The number in circles indicated variants counts occurred in individuals. (**B)** Analysis of 6 STR loci surrounding *BRCA1*. A total of 34 HOC patients who harbored recurrent *BRCA1* variants with DNA available were analyzed. The percentage of individuals with matching haplotypes was noted on the right side. The primary and secondary haplotypes were denoted in red and blue, respectively. The patients with *BRCA1*:c.5470-5477del has been reported in our previous study as a founder mutation in Han Chinese^11^.

**
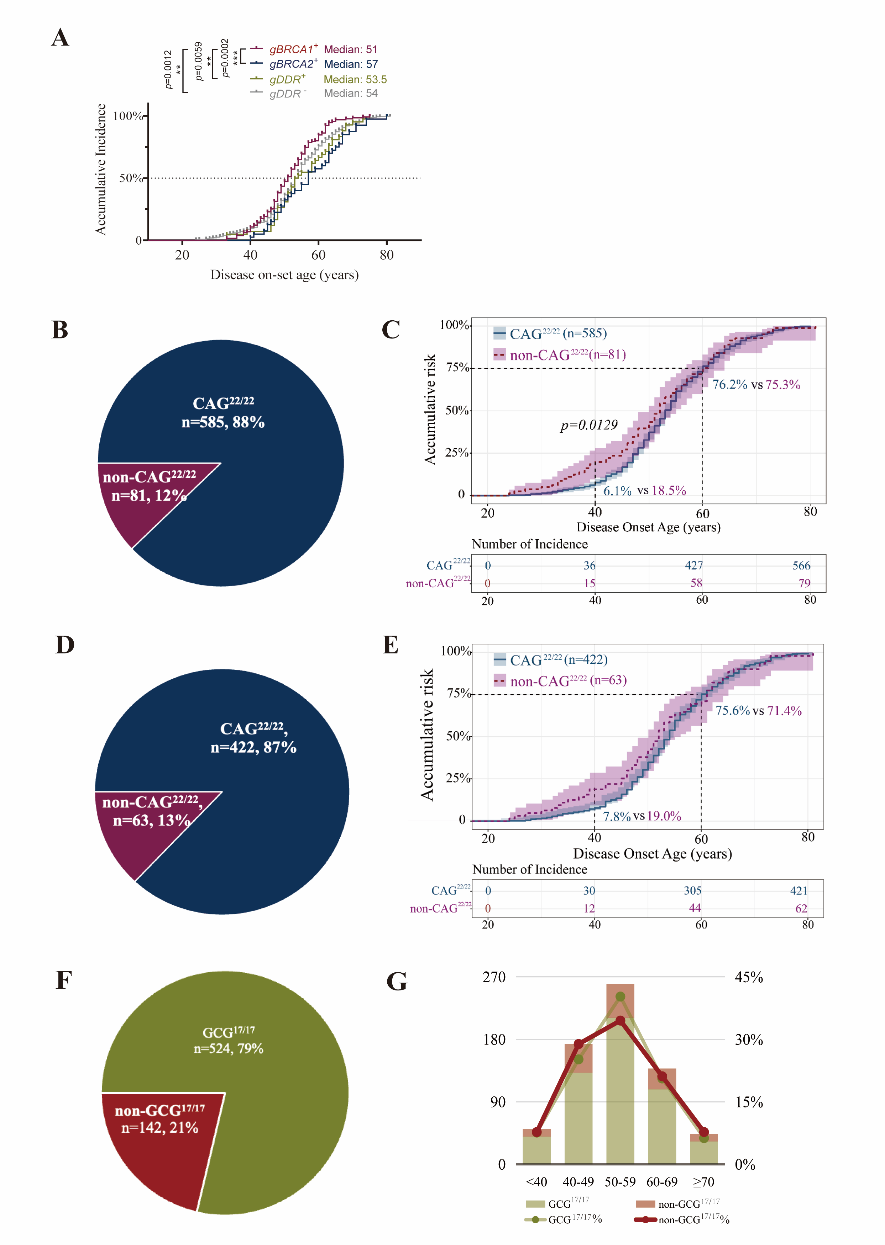
**

**Figure S2.** Disease onset age and cumulative analysis incidence by *AR*-CAG and *AR*-GCG polymorphisms in Henan ovarian cancer (HOC) patients.

**(A**) Accumulative incidence of OC for *BRCA1*^+^ (n=139, red), *BRCA2*^+^ (n=43, blue), DDR^+^ (n=51, green), and DDR^-^ (n=443, gray) HOC patients. Disease onset age of the patients with different genetic predispositions were present as median with range and compared using the log-rank test. (**B**) Proportion of HOC patients with the *AR*-CAG^22/22^ (n=585, blue), or *AR* non-CAG^22/22^ (n=81, purple) genotype. (**C)** Cumulative disease incidence of HOC patients with *AR*-CAG^22/22^ (n=585, blue), non-CAG^22/22^ (n=81, purple) genotype. The log-rank test was used to compared the difference in disease onset age between *AR*-CAG^22/22^ and non-CAG^22/22^ genotype. Accumulative disease onset risk of *AR*-CAG^22/22^ and non-CAG^22/22^ patients are indicated. (**D**). Proportion of *BRCA1/2*^-^ HOC patients with the *AR*-CAG^22/22^ (n=422, blue), or *AR* non-CAG^22/22^ (n=63, red) genotype. **(E)** *AR*-CAG^22/22^ and *AR* non-CAG^22/22^ accumulative incidence. The log-rank test was performed for patients with disease onset age younger than 60. **(F)** Proportion of total HOC patients with the *AR*-GCG^17/17^ (n=524, green), or *AR* non-GCG^17/17^ (n=142, red) genotype. **(G)** The number (left Y-axis) and proportion (right Y-axis) of patients with *AR* GCG^17/17^ or non-GCG^17/17^ genotypes in different ages.

**Supplementary Tables**

**Table S1.** Genes included in the DNA damage response (DDR)-targeted panel of this study.

| Function | Gene name |
| --- | --- |
| Homologous Recombination Repair | *ATM, BARD1, BRCA1, BRCA2, FAM175A, GEN1, RAD51B, RAD51D, RAD54L* |
| Mismatch Repair | *MLH1, MLH3, MSH2, MSH6, PMS2* |
| Non-homologous end-joining | *MRE11A, NBN, RAD50* |
| Base excision repair | *MUTYH* |
| Nucleotide excision repair | *ERCC3* |
| Fanconi Anemia | *BRIP1, FANCA, FANCL, FANCM, PALB2, RAD51C* |
| Others | *APC, AR, ATR, CDH1, CDK12, CHEK1, CHEK2, ESR1, EPCAM, HDAC2, HER2, NF1, PIK3CA, PPP2R2A, PPM1D, PTEN, STK11, TP53, TSC1, TSC2* |

**Table S2.** Total of 232 pathogenic or likely pathogenic variants (P/LPVs) in DNA damage response (DDR) genes.

Data was shown in Supplementary file 2.xls

**Table S3.** Clinical characteristics of Henan ovarian cancer (HOC) patients with double pathogenic or likely pathogenic variants (P/LPVs).

| Case No. | Pathology Diagnosis | Onset age (years) | PS | FH | Variants | Biological Function |
| --- | --- | --- | --- | --- | --- | --- |
| 1 | HGSC | 40 | BC | Y | *BRCA1*:c.3228_3229del/p.Gly1077fs  *RAD51D*: c.694>T/p.Arg232* | HRD |
| 2 | Unspecific | 44 | DLBCL | N | *BRCA2*:c.5789del/p.Leu1930fs  *MRE11A*:c.1336-2A>G | HRD |
| 3 | HGSC | 48 | BC | N | *BRCA1*:c.5521del/p.Ser1841fs  *MRE11A*:c.1876+1del | HRD |
| 4 | HGSC | 51 | N | N | *BRCA1*:c.5470_5477del/p.Ile1824fs  *ATM*:c.8987+1G>T | HRD |
| 5 | HGSC | 54 | N | N | *BRCA1*:c.3906del/p.Glu1302fs  *GEN1*:c.1201C>T/p.Arg401* | HRD |
| 6 | HGSC | 54 | N | N | *BRCA1*:c.1660G>T/p.Glu554*  *MUTYH*:c.857G>A:p.Gly286Glu | HRD+BED |
| 7 | HGSC | 55 | N | N | *BRCA2*:c.7043del/p.Asn2348fs  *MUTYH*:c.848del/p.Met283fs | HRD+BED |
| 8 | HGSC | 53 | BC | N | *BRCA1*:c.5470_5477del/p.Ile1824fs  *MUTYH*:c.857G>A/p.Gly286Glu | HRD+BED |
| 9 | HGSC | 60 | N | N | *BRCA1*:c.3859del/p.Glu1287fs  *FANCA*: c.1A>G/p.Met1? | HRD+FA |
| 10 | HGSC | 64 | N | Y | *BRCA1*:c.3294del/p.Pro1099fs  *BRCA2*: c.3666del/p.His1223fs | HRD |

Note: HGSC, high-grade serous carcinoma; PS, personal history; BC, breast cancer;DLBCL, Diffuse Large B-Cell Lymphoma; FH, family history; HRD, homologous recombination deficiency; BED, base excision deficiency; FA, Fanconi anemia.

**Table S4.** Ovarian cancer risk estimates for other DNA damage response genes in the Henan ovarian cancer (HOC) cohort

| Gene list | HOC (n=666) | |  | gnomAD v4.1.0 east Asian(n=22448) | | | | |  | ChinaMAP v2020-03.beta (n=10588) | | | | |
| --- | --- | --- | --- | --- | --- | --- | --- | --- | --- | --- | --- | --- | --- | --- |
|  | cases | % |  | cases | % | OR | 95% CI | *p*-value |  | cases | % | OR | 95% CI | *p*-value |
| *ERCC3* | 1 | 0.15% |  | 4 | 0.02% | 8.4 | 0.2 - 85.3 | 0.14 |  | 0 | 0.00% | Inf | 0.4 - Inf | 0.06 |
| *CHEK1* | 1 | 0.15% |  | 0 | 0.00% | Inf | 0.9 - Inf | 0.03 |  | 0 | 0.00% | Inf | 0.4 - Inf | 0.06 |
| *RAD54L* | 1 | 0.15% |  | 4 | 0.02% | 8.4 | 0.2 - 85.3 | 0.14 |  | 19 | 0.18% | 0.8 | 0.02 - 5.3 | 1.00 |
| *FANCL* | 1 | 0.15% |  | 5 | 0.02% | 6.7 | 0.1 - 60.4 | 0.16 |  | 5 | 0.05% | 3.2 | 0.07 - 28.4 | 0.31 |
| *TP53* | 1 | 0.15% |  | 7 | 0.03% | 4.8 | 0.1 - 37.6 | 0.21 |  | 2 | 0.02% | 7.9 | 0.1 - 152.7 | 0.17 |
| *FANCM* | 1 | 0.15% |  | 8 | 0.04% | 4.2 | 0.1 - 31.5 | 0.23 |  | 7 | 0.07% | 2.3 | 0.05 - 17.7 | 0.39 |
| *MSH6* | 2 | 0.30% |  | 21 | 0.09% | 3.2 | 0.4- 13.2 | 0.14 |  | 10 | 0.09% | 3.2 | 0.3 - 15.0 | 0.16 |
| *ATM* | 3 | 0.45% |  | 46 | 0.20% | 2.2 | 0.4 - 6.9 | 0.17 |  | 20 | 0.19% | 2.4 | 0.5 - 8.1 | 0.15 |
| *FANCA* | 3 | 0.45% |  | 48 | 0.21% | 2.1 | 0.4 - 6.6 | 0.18 |  | 20 | 0.19% | 2.4 | 0.5 - 8.1 | 0.15 |
| *PALB2* | 1 | 0.15% |  | 22 | 0.10% | 1.5 | 0.04 - 9.5 | 0.49 |  | 11 | 0.10% | 1.4 | 0.03 - 10.0 | 0.52 |
| *GEN1* | 3 | 0.45% |  | 72 | 0.32% | 1.4 | 0.3 - 4.3 | 0.48 |  | 19 | 0.18% | 2.5 | 0.5 - 8.6 | 0.14 |
| *ATR* | 1 | 0.15% |  | 39 | 0.17% | 0.9 | 0.02 - 5.1 | 1 |  | 9 | 0.09% | 1.8 | 0.04 - 12.8 | 0.46 |

Note: Fisher’s exact test was applied to determine relative risk for each gene by comparing to their prevalence in the gnomAD (east Asian, v4.1.0) and ChinaMAP databases (v2020-03.beta). OR: odds ratio; 95% CI: 95% confidence interval.

**Table S5.** Prevalence of ovarian cancer predisposition genes across different populations.

| **Gene** | HOC cohort (n=666) | |  | Harter et al.^21^ (n=473) | |  | Norquist et al.^22^ (n=1915) | |  | Pennington et al.^23^ (n=367) | |
| --- | --- | --- | --- | --- | --- | --- | --- | --- | --- | --- | --- |
|  | Prevalence | cases |  | Prevalence | cases |  | Prevalence | cases |  | Prevalence | cases |
| *BRCA1* | 20.87% | 139 |  | 15.86% | 75 |  | 9.50% | 182 |  | 13.35% | 49 |
| *BRCA2* | 6.46% | 43 |  | 4.86% | 23 |  | 5.12% | 98 |  | 4.63% | 17 |
| *BARD1* | 0.00% | 0 |  | 0.00% | 0 |  | 0.21% | 4 |  | 0.54% | 2 |
| *RAD51D* | 1.65% | 11 |  | 0.63% | 3 |  | 0.57% | 11 |  | 1.09% | 4 |
| *BRIP1* | 0.45% | 3 |  | 0.42% | 2 |  | 1.36% | 26 |  | 1.09% | 4 |
| *RAD51C* | 0.45% | 3 |  | 2.75% | 13 |  | 0.57% | 11 |  | 0.82% | 3 |
| *PALB2* | 0.15% | 1 |  | 0.85% | 4 |  | 0.63% | 12 |  | 0.54% | 2 |
| *MSH2* | 0.30% | 2 |  | 0.00% | 0 |  | 0.00% | 0 |  | 0.27% | 1 |
| *MSH6* | 0.30% | 2 |  | 0.00% | 0 |  | 0.16% | 3 |  | 0.27% | 1 |
| *RAD50* | 0.60% | 4 |  | 0.21% | 1 |  | 0.16% | 3 |  | 0.27% | 0 |
| *CHEK2* | 0.00% | 0 |  | 0.42% | 2 |  | 0.57% | 11 |  | 0.82% | 3 |
| *ATM* | 0.45% | 3 |  | 0.42% | 2 |  | 0.57% | 11 |  | 0.00% | 0 |
| *FAM175A* | 0.00% | 0 |  | 0.21% | 1 |  | 0.16% | 3 |  | 0.54% | 2 |
| *MRE11A* | 0.30% | 2 |  | 0.42% | 2 |  | 0.10% | 2 |  | 0.00% | 0 |
| *TP53* | 0.15% | 1 |  | 0.00% | 0 |  | 0.31% | 6 |  | 0.82% | 3 |
| *NBN* | 0.00% | 0 |  | 0.42% | 2 |  | 0.47% | 9 |  | 0.27% | 1 |
| Total | **32.13%** | **214** |  | **27.48%** | **130** |  | **20.47%** | **392** |  | **25.07%** | **92** |

Note: The table includes only 16 genes common to all studies referenced above.

**Table S6.** Clinical characteristics of patients from previous Chinese studies on genetic predisposition to ovarian cancer.

|  | | *Li et al.* 2021^11^ (n=530) | | |  | *Wen et al.* 2023^31^ (n=945) | |  | *Li et al.* 2018^30^ (n=1331) | |  | *Shi et al.* 2017^29^ (n=916) | |  | *Wu et al.* 2017^32^ (n=826) | |
| --- | --- | --- | --- | --- | --- | --- | --- | --- | --- | --- | --- | --- | --- | --- | --- | --- |
|  |  | cases | % |  | | cases | % |  | cases | % |  | cases | % |  | cases | % |
| Genetic  predispositions | *BRCA1^+^* | 117 | 22.08% |  | | 110 | 11.64% |  | 227 | 17.05% |  | 140 | 15.28% |  | 172 | 20.82% |
|  | *BRCA2^+^* | 34 | 6.42% |  | | 36 | 3.81% |  | 70 | 5.26% |  | 57 | 6.22% |  | 63 | 7.63% |
|  | *BRCA1/2^-^* | 380 | 71.70% |  | | 799 | 84.55% |  | 1034 | 77.69% |  | 722 | 78.82% |  | 591 | 71.55% |
| Disease  on-set age | ≤40 years | 52 | 9.81% |  | | na |  |  | 143 | 10.74% |  | 66 | 7.21% |  | na |  |
|  | ＞40years | 478 | 90.19% |  | | na |  |  | 1120 | 84.15% |  | 850 | 92.79% |  | na |  |
|  | unknown | 0 | 0.00% |  | | na |  |  | 68 | 5.11% |  | 0 | 0.00% |  | 2 | 0.24% |
| Stages of disease | stage I/II | 94 | 17.74% |  | | 164 | 17.35% |  | 283 | 21.26% |  | 63 | 6.88% |  | 117 | 14.16% |
|  | stage Ⅲ/Ⅳ | 378 | 71.32% |  | | 726 | 76.83% |  | 588 | 44.18% |  | 491 | 53.60% |  | 689 | 83.41% |
|  | unknown | 58 | 10.94% |  | | 55 | 5.82% |  | 460 | 34.56% |  | 362 | 39.52% |  | 20 | 2.42% |
| Histological  subtypes | SC | 429 | 80.94% |  | | na |  |  | 843 | 63.34% |  | 726 | 79.26% |  | 619 | 74.94% |
|  | Non-SC | 46 | 8.68% |  | | na |  |  | 371 | 27.87% |  | 189 | 20.63% |  | 204 | 24.70% |
|  | unknown | 55 | 10.38% |  | | na |  |  | 117 | 8.79% |  | 1 | 0.11% |  | 3 | 0.36% |
| Family  History | yes | 50 | 9.43% |  | | na |  |  | 106 | 7.96% |  | 174 | 19.00% |  | 96 | 11.62% |
|  | no | 480 | 90.57% |  | | na |  |  | 1148 | 86.25% |  | 742 | 81.00% |  | 730 | 88.38% |
|  | unknown | 0 | 0.00% |  | | na |  |  | 77 | 5.79% |  | 0 | 0.00% |  | 0 | 0.00% |

Note: SC, serous carcinoma; na, not available.

**Table S7.** Recurrent *BRCA1* pathogenic or likely pathogenic variants (P/LPVs) identified in studies of Chinese ovarian cancer patients.

| Codon changes  of *BRCA1* | HOC cohort,  *BRCA1*^+^=139 | |  | *Wen et al.* 2023^31^,  *BRCA1*^+^=110 | |  | *Li et al.* 2018^30^,  *BRCA1*^+^=225 | |  | *Shi et al.* 2017^29^,  *BRCA1*^+^=120 | |  | *Wu et al. 2017*^32^,  *BRCA1*^+^=227 | |
| --- | --- | --- | --- | --- | --- | --- | --- | --- | --- | --- | --- | --- | --- | --- |
|  | cases | % |  | cases | % |  | cases | % |  | cases | % |  | cases | % |
| c.5470_5477del | 11 | 7.9% |  | 7 | 6.4% |  | 18 | 8.0% |  | 10 | 8.3% |  | 11 | 4.8% |
| c.981_982del | 8 | 5.8% |  |  |  |  | 9 | 4.0% |  | 6 | 5.0% |  | 9 | 4.0% |
| c.4065_4068del | 6 | 4.3% |  |  |  |  |  |  |  | 4 | 3.3% |  | 5 | 2.2% |
| c.3442del |  |  |  |  |  |  | 4 | 1.8% |  | 3 | 2.5% |  | 4 | 1.8% |
| c.1465G>T | 3 | 2.2% |  |  |  |  | 3 | 1.3% |  |  |  |  | 3 | 1.3% |
| c.3770_3771del |  |  |  |  |  |  | 7 | 3.1% |  | 6 | 5.0% |  |  |  |
| c.2110_2111del |  |  |  |  |  |  | 5 | 2.2% |  |  |  |  | 3 | 1.3% |
| c.3607C>T | 3 | 2.2% |  | 3 | 2.7% |  |  |  |  |  |  |  |  |  |
| c.3294del | 5 | 3.6% |  |  |  |  | 3 | 1.3% |  |  |  |  |  |  |
| c.5521del | 4 | 2.9% |  |  |  |  | 4 | 1.8% |  |  |  |  |  |  |
| c.2572C>T | 4 | 2.9% |  |  |  |  |  |  |  |  |  |  | 5 | 2.2% |
| In total | 44 | 31.7% |  | 10 | 9.1% |  | 53 | 23.6% |  | 29 | 24.2% |  | 40 | 17.6% |

Note: Only the recurrent PVs/LPVs in *BRCA1* presenting in at least two different studies were included in this table
